# Vaccinia virus B5 protein affects the glycosylation, localization and stability of the A34 protein

**DOI:** 10.1099/vir.0.020677-0

**Published:** 2010-07

**Authors:** Adrien Breiman, Geoffrey L. Smith

**Affiliations:** Department of Virology, Faculty of Medicine, Imperial College London, St Mary’s Campus, Norfolk Place, London W2 1PG, UK

## Abstract

Vaccinia virus has two infectious forms, the intracellular mature virus, which has a single envelope, and the extracellular enveloped virus (EEV), which is surrounded by two lipid bilayers. The outer membrane of the EEV contains at least six viral proteins. Among them A34, a type II membrane glycoprotein, and B5, a type I membrane glycoprotein, form a complex and are involved in processes such as morphogenesis and EEV entry. A34 is required for normal incorporation of B5 into the EEV membrane. Here, we used a virus lacking B5 and viruses with mutations in the B5 membrane-proximal stalk region and looked at the effect of those modifications on A34. Data presented show that B5 is required for the correct glycosylation, trafficking and stability of A34, emphasizing the complex interactions and mutual dependence of these vaccinia EEV proteins.

*Vaccinia virus* (VACV) is the prototypical member of the genus *Orthopoxvirus* of the *Poxviridae.* It replicates in the cytosol and produces multiple types of infectious virions (Smith *et al.*, 2002; Condit *et al.*, 2006; Roberts & Smith, 2008). The first infectious progeny is the intracellular mature virus (IMV), which is surrounded by a single-lipid envelope (Dales & Siminovitch, 1961; Hollinshead *et al.*, 1999) and remains in the cell until cell lysis. However, some IMV are transported via microtubules to the early endosomes or *trans*-Golgi network where they are wrapped by two cellular membranes containing several VACV proteins. The resulting intracellular enveloped virus (IEV) is then transported on microtubules to the cell surface where the outer membrane fuses with the plasma membrane to externalize a double-enveloped virus by exocytosis. This virion is called cell-associated enveloped virus (CEV) if it remains on the cell surface, or extracellular enveloped virus (EEV) if it is released from the cell. The CEV/EEV outer membrane contains at least six viral proteins: A33 (Roper *et al.*, 1996), A34 (Duncan & Smith, 1992), A56 (Shida, 1986), B5 (Engelstad *et al.*, 1992; Wolffe *et al.*, 1993), F13 (Blasco & Moss, 1991) and K2 (Turner & Moyer, 2006; Wagenaar & Moss, 2007). A34 is a type II transmembrane protein with different glycoforms between 23 and 28 kDa and its extracellular part contains a C-type lectin-like domain (Duncan & Smith, 1992). A K151D point mutation in the VACV strain Western Reserve (WR) A34, which is present naturally in the VACV International Health Department (IHD)-J strain, caused an increase in EEV release (Blasco *et al.*, 1993). Similarly, deletion of the *A34R* gene (vΔA34R) from VACV WR caused a 25-fold increase in EEV, but such EEV had a fivefold reduction in specific infectivity (McIntosh & Smith, 1996). Deletion or suppression of the *A34R* gene caused a small plaque phenotype (Duncan & Smith, 1992; McIntosh & Smith, 1996), inability to form actin tails (Wolffe *et al.*, 1997; Sanderson *et al.*, 1998) and severe attenuation (McIntosh & Smith, 1996).

B5 is a 42 kDa type I transmembrane glycoprotein (Engelstad *et al.*, 1992; Isaacs *et al.*, 1992) with an extracellular domain composed of four short consensus repeats (SCRs) characteristic of complement control proteins (Takahashi-Nishimaki *et al.*, 1991), although there is no evidence that B5 regulates complement activity. After the SCRs B5 has an acidic stalk region (ST) before the transmembrane domain (TM) and a short cytoplasmic tail (CT). Both the SCRs and CT are dispensable for targeting B5 to the EEV membrane (Herrera *et al.*, 1998; Lorenzo *et al.*, 1998; Mathew *et al.*, 1998), although the latter affects its transport to the cell surface (Mathew *et al.*, 2001) and recycling via endosomes (Ward & Moss, 2000). B5 is needed for IMV wrapping to form IEV (Engelstad & Smith, 1993; Wolffe *et al.*, 1993). B5 and A34 interact (Rottger *et al.*, 1999; Earley *et al.*, 2008; Perdiguero *et al.*, 2008; Roberts *et al.*, 2009) and in the absence of A34, the amount of B5 incorporated in EEV is decreased markedly (Earley *et al.*, 2008; Perdiguero *et al.*, 2008; Roberts *et al.*, 2009). B5 and A34 each affect the glycosaminoglycan (GAG)-dependent rupture of the EEV outer membrane during EEV entry (Law *et al.*, 2006; Roberts *et al.*, 2009).

Although B5 expressed on its own displays a cellular localization profile very similar to the one observed in the context of viral infection (Katz *et al.*, 1997; Lorenzo *et al.*, 2000), this is not the case for A34. In infected cells, A34 is found at the Golgi, on the cell surface and in CEV/EEV. In contrast, when it is expressed alone it accumulates in the perinuclear region and does not go to the plasma membrane (Lorenzo *et al.*, 2000). In addition, attempts to express A34 on its own from classical eukaryotic expression vectors (pcDNA3, pCI) and in several recombinant expression systems yielded poor levels of expression. For example, in a system where soluble forms of EEV proteins A56, B5 and A33 were expressed in CHO cells and secreted into the medium, the yield obtained for A34 was about 20-fold lower than for B5 (Law *et al.*, 2005; Pütz *et al.*, 2005; M. Law unpublished data). Here, we present data showing that in the absence of B5, the level of A34 is markedly decreased, most probably because of misfolding and consequential degradation.

In a recent study on EEV entry, we generated several VACV mutants with alterations in the B5 stalk acidic residues (Roberts *et al.*, 2009), for the structure of these mutants see Fig. 1(a). Using those viruses, we analysed lysates from infected RK13 cells by immunoblotting with mouse monoclonal antibodies (mAbs) against B5 (36-6; Roberts *et al.*, 2009), A34 (34-1; Roberts *et al.*, 2009) and the IMV protein D8 (AB1.1; Parkinson & Smith, 1994) as an infection control (Fig. 1b). Mouse mAbs against the A34 and B5 proteins were produced by immunization of mice with purified recombinant protein expressed from mammalian cells (Law *et al.*, 2005; Pütz *et al.*, 2005). As noted previously, these mutations affected the electrophoretic mobility of B5 (Roberts *et al.*, 2009). In addition, this analysis showed that in the absence of B5, the amount of A34 in the infected cells was reduced considerably, and with some of the B5 mutants the glycosylation profile of A34 was different. When B5 was deleted, one distinct band at about 20 kDa (A34*) was observed instead of the 23–28 kDa bands made by wild-type (WT) virus. In contrast, deletion of all the B5 SCRs (vSCR0), had no effect and A34 retained the WT profile (note the remaining B5 fragment was not visible due to its small size). However, substitution of the acidic residues of the stalk with alanines (vST2-35ala) had the same effect as deleting B5. Moreover, substitution of the five acidic residues closest to the membrane (vST23-35ala) also led to the A34* profile. Interestingly, vST2-16 and vST28-35 showed a mixed profile with the A34* band evident together with higher molecular mass forms (Fig. 1b). Since vST23-35ala and vST28-35ala differ only at residue 23 and display distinct A34 glycosylation profiles, we wondered if that amino acid could by itself influence A34 glycosylation. To address this, a recombinant VACV in which the aspartic acid 23 of the stalk region was mutated to alanine (vST23ala) was constructed by using transient dominant selection as described previously (Roberts *et al.*, 2009). When tested by immunoblotting as above, vST23ala showed the same A34 profile as the WT virus and therefore, mutating the aspartic acid 23 is not sufficient to alter A34 glycosylation (Fig. 1c). Overall, analysis of these mutants suggested that the B5 stalk is important for correct glycosylation of A34.

To determine more precisely the nature of the A34 20 kDa isoform, we used drugs that affect glycosylation: namely tunicamycin, an inhibitor of *N*-acetylglucosamine transferase and kifunensin, an inhibitor of *α*-mannosidase I (Fig. 2a). RK13 cells were infected with WR or vΔB5R at 5 p.f.u. per cell for 90 min and then incubated overnight in Dulbecco’s modified Eagle’s medium containing 2.5 % fetal bovine serum with or without 1 μM tunicamycin or 5 μM kifunensin. Cell lysates were then prepared and analysed by immunoblotting (Fig. 2b). Treatment of WR-infected cells with tunicamycin produced a single A34 band, corresponding to the unglycosylated polypeptide (A34_ug_). The A34_ug_ is predicted to be 19.6 kDa (Duncan & Smith, 1992), and we observed a slightly smaller band of about 17 kDa. In vΔB5R-infected cells, A34 is slightly larger than A34_ug_ and was still reduced in size in the presence of tunicamycin, indicating that A34* is a partially glycosylated form. Notably, levels of B5 and A34 both decreased in the presence of tunicamycin, indicating that glycosylation is required for stability of these proteins. In the presence of kifunensin, the A34* pattern was observed in WR-infected cells, suggesting that A34* represents an intermediate with nine mannose residues (Man_9_), before trimming by the *α*-mannosidases.

Another interesting observation was that the amount of A34 in the absence of B5 was increased by kifunensin treatment. This is in agreement with reports showing that processing by *α*-mannosidases acts as a signal to target misfolded proteins for proteasomal degradation and that inhibition of these enzymes by kifunensin treatment leads to accumulation of misfolded (Man_9_)-glycoproteins (Olivari & Molinari, 2007). To address this further, the stability of A34 with time was investigated (Fig. 2c). RK13 cells were infected with WR or vΔB5R as before and cell lysates were prepared at 4, 8, 12 and 24 h p.i. and analysed by immunoblotting. Up to 8 h p.i., A34 was easily detected in cells infected by either virus, although it was less abundant in vΔB5R-infected cells, but thereafter A34 declined substantially in vΔB5R-infected cells and was barely visible at 24 h. This suggests that synthesis and accumulation of A34 starts normally without B5, but as the rate of synthesis decreases later during infection, the level of A34 declines. Collectively, those data suggest that in the absence of B5, A34 is misfolded and degraded.

Next, the effect of A34 glycosylation status on the subcellular localization was investigated. BSC-1 cells were infected with viruses at 2 p.f.u. per cell for 8 h, fixed with PBS–4 % paraformaldehyde (PFA) for 10 min on ice and then in PBS–8 % PFA for 20 min at room temperature. Fixed cells were permeabilized with 0.2 % Triton X-100 and incubated with anti-A34 mAb and a rabbit anti-protein disulphide isomerase Ab (anti-PDI; Abcam) to stain the endoplasmic reticulum (Fig. 3). Consistent with Fig. 2(c), significant levels of A34 were present in both WR- and vΔB5R-infected cells at 8 h p.i. In WR-infected cells, the anti-A34 mAb labelled the Golgi as well as punctate structures corresponding to virions in the periphery, as described previously (Lorenzo *et al.*, 2000), but no significant co-localization with PDI was observed (Fig. 3a). In contrast, in vΔB5R-infected cells A34 was present throughout the cell in a reticular pattern co-localizing with PDI (Fig. 3a), similar to that seen when A34 was expressed from a Semliki Forest virus vector (Lorenzo *et al.*, 2000). No staining of VACV particles was observed, and this may be explained by the wrapping defect of vΔB5R (Engelstad & Smith, 1993; Wolffe *et al.*, 1993). Cells infected with vΔB5R, vST23-35ala and vST28-35ala all showed a significant amount of A34 in the ER and nuclear envelope, but vST23-35ala and vST28-35ala also showed some staining of viral particles (Fig. 3b; enlargement of the punctate staining representing virions is shown for the vST28-35 image). Cells infected with vST23-35ala generally had fewer particles than WR- or even vST28-35ala-infected cells. This is consistent with the fact that vST23-35 produced much less EEV than WR, whereas EEV production by vST28-35ala was only slightly reduced (Roberts *et al.*, 2009). Overall, these data show that the A34* band in SDS-PAGE correlates with the presence of A34 in the ER. This is consistent with A34* being a (Man_9_)-A34 that accumulates in the ER.

Data presented here indicate that in the absence of B5, or in presence of some mutated forms of B5, A34 is not correctly folded and accumulates in the ER as a partially glycosylated intermediate. Ultimately, at least in the case of vΔB5R, this would lead to proteasomal degradation. A hypothesis to explain these data would be that an interaction of the negatively charged acidic residues in the B5 stalk region with positive charges of A34 might help A34 to acquire the correct conformation. Alternatively, the B5 stalk could play a role in the trafficking of the B5/A34 complex.

Taken together with the previous data showing that A34 is required for proper incorporation of B5 in the EEV membrane, this shows that there is a complex mutual interaction between these two VACV proteins making it difficult to unravel their respective roles in virus wrapping, egress and re-entry due to their inter-dependence.

## Figures and Tables

**Fig. 1.**
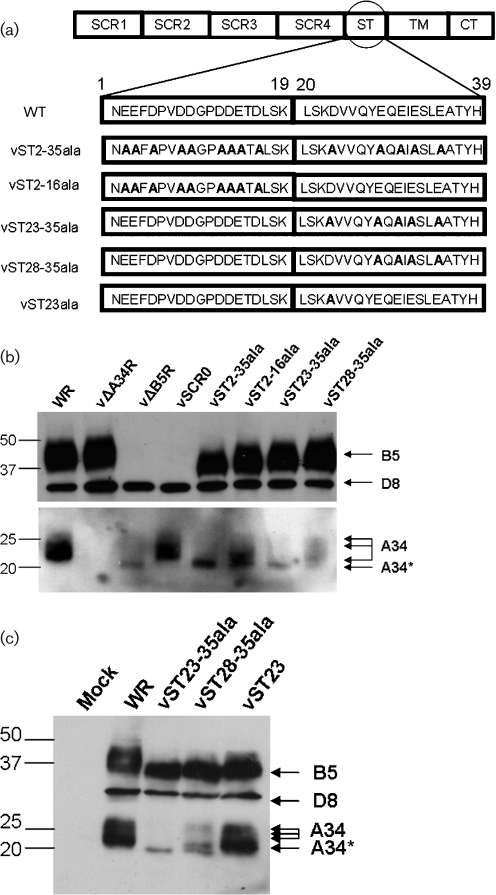
B5 affects the abundance and size of A34. (a) Structure of the B5 stalk mutants used in this study. The amino acid residues of the stalk are numbered 1–39 and modified residues are shown in bold. (b, c). RK13 cells were infected with the indicated viruses and cell lysates were prepared at 16 h post-infection (p.i.) and immunoblotted with mAbs against B5, D8 and A34. The positions of molecular mass markers are shown on the left in kDa.

**Fig. 2.**
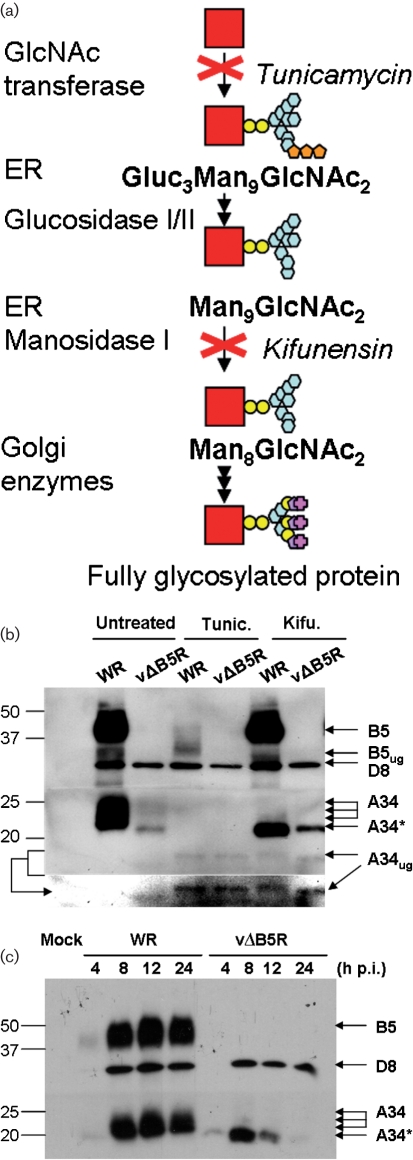
In the absence of B5, A34 has aberrant glycosylation and is targeted for degradation. (a) Simplified diagram of the glycosylation pathway showing where tunicamycin and kifunensin act. Gluc, Glucose; GlcNAc, *N*-acetylglucosamine; Man, mannose. (b) RK13 cells were infected with WR and vΔB5R and incubated with or without tunicamycin (Tunic.) or kifunensin (Kifu.). At 16 h p.i., cell lysates were prepared and analysed by immunoblotting with anti-B5, anti-D8 and anti-A34 mAbs. Another image of the bottom section of the membrane is shown for clarity. A34_ug_ and B5_ug_, unglycosylated A34 and unglycosylated B5. (c) A34 is synthesized in the absence of B5, but is degraded over time. RK13 cells were infected with WR or vΔB5R and harvested at the indicated times p.i. Cell lysates were prepared and analysed by immunoblotting as in (b). The positions of molecular mass markers are shown on the left in kDa.

**Fig. 3.**
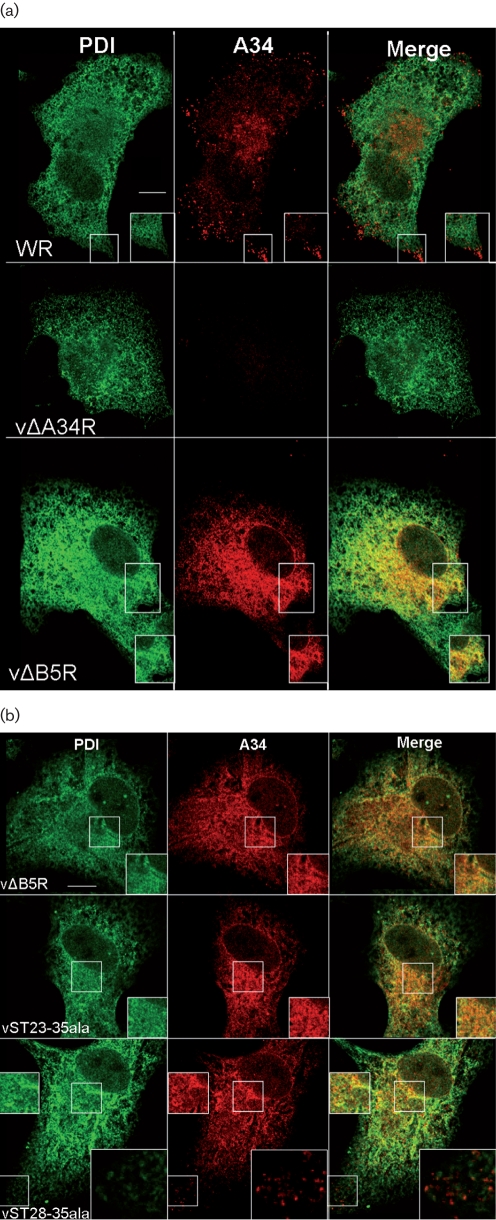
Absence or alteration of B5 leads to accumulation of A34 in the ER. (a, b). BSC-1 cells were infected for 8 h with the viruses shown, fixed and processed for immunofluorescence using anti-A34 mAb followed by anti-mouse-Alexa 546 (red) and anti-PDI followed by anti-rabbit-Alexa 488 (green). Samples were viewed on a Zeiss 510 Meta confocal microscope using Zeiss LSM software. The right panel of each row shows the merged image of the left and centre panel. Boxes within individual panels show regions of the cell before and after magnification. Bars, 10 μM.

## References

[r1] Blasco, R. & Moss, B. (1991). Extracellular vaccinia virus formation and cell-to-cell virus transmission are prevented by deletion of the gene encoding the 37,000-Dalton outer envelope protein. J Virol 65, 5910–5920. 1920620 10.1128/jvi.65.11.5910-5920.1991 PMC250254

[r2] Blasco, R., Sisler, J. R. & Moss, B. (1993). Dissociation of progeny vaccinia virus from the cell membrane is regulated by a viral envelope glycoprotein: effect of a point mutation in the lectin homology domain of the A34R gene. J Virol 67, 3319–3325. 8497053 10.1128/jvi.67.6.3319-3325.1993 PMC237674

[r3] Condit, R. C., Moussatche, N. & Traktman, P. (2006). In a nutshell: structure and assembly of the vaccinia virion. Adv Virus Res 66, 31–124. 16877059 10.1016/S0065-3527(06)66002-8

[r4] Dales, S. & Siminovitch, L. (1961). The development of vaccinia virus in Earle’s L strain cells as examined by electron microscopy. J Biophys Biochem Cytol 10, 475–503. 13719413 10.1083/jcb.10.4.475 PMC2225098

[r5] Duncan, S. A. & Smith, G. L. (1992). Identification and characterization of an extracellular envelope glycoprotein affecting vaccinia virus egress. J Virol 66, 1610–1621. 1738204 10.1128/jvi.66.3.1610-1621.1992 PMC240895

[r6] Earley, A. K., Chan, W. M. & Ward, B. M. (2008). The vaccinia virus B5 protein requires A34 for efficient intracellular trafficking from the endoplasmic reticulum to the site of wrapping and incorporation into progeny virions. J Virol 82, 2161–2169. 18094183 10.1128/JVI.01971-07 PMC2258950

[r7] Engelstad, M. & Smith, G. L. (1993). The vaccinia virus 42-kDa envelope protein is required for the envelopment and egress of extracellular virus and for virus virulence. Virology 194, 627–637. 8503178 10.1006/viro.1993.1302

[r8] Engelstad, M., Howard, S. T. & Smith, G. L. (1992). A constitutively expressed vaccinia gene encodes a 42-kDa glycoprotein related to complement control factors that forms part of the extracellular virus envelope. Virology 188, 801–810. 1585649 10.1016/0042-6822(92)90535-w

[r9] Herrera, E., Lorenzo, M. M., Blasco, R. & Isaacs, S. N. (1998). Functional analysis of vaccinia virus B5R protein: essential role in virus envelopment is independent of a large portion of the extracellular domain. J Virol 72, 294–302. 9420227 10.1128/jvi.72.1.294-302.1998 PMC109376

[r10] Hollinshead, M., Vanderplasschen, A., Smith, G. L. & Vaux, D. J. (1999). Vaccinia virus intracellular mature virions contain only one lipid membrane. J Virol 73, 1503–1517. 9882356 10.1128/jvi.73.2.1503-1517.1999 PMC103975

[r11] Isaacs, S. N., Wolffe, E. J., Payne, L. G. & Moss, B. (1992). Characterization of a vaccinia virus-encoded 42-kilodalton class I membrane glycoprotein component of the extracellular virus envelope. J Virol 66, 7217–7224. 1433514 10.1128/jvi.66.12.7217-7224.1992 PMC240424

[r12] Katz, E., Wolffe, E. J. & Moss, B. (1997). The cytoplasmic and transmembrane domains of the vaccinia virus B5R protein target a chimeric human immunodeficiency virus type 1 glycoprotein to the outer envelope of nascent vaccinia virions. J Virol 71, 3178–3187. 9060681 10.1128/jvi.71.4.3178-3187.1997 PMC191450

[r13] Law, M., Putz, M. M. & Smith, G. L. (2005). An investigation of the therapeutic value of vaccinia-immune IgG in a mouse pneumonia model. J Gen Virol 86, 991–1000. 15784892 10.1099/vir.0.80660-0

[r14] Law, M., Carter, G. C., Roberts, K. L., Hollinshead, M. & Smith, G. L. (2006). Ligand-induced and nonfusogenic dissolution of a viral membrane. Proc Natl Acad Sci U S A 103, 5989–5994. 16585508 10.1073/pnas.0601025103 PMC1424662

[r15] Lorenzo, M. M., Herrera, E., Blasco, R. & Isaacs, S. N. (1998). Functional analysis of vaccinia virus B5R protein: role of the cytoplasmic tail. Virology 252, 450–457. 9878625 10.1006/viro.1998.9483

[r16] Lorenzo, M. M., Galindo, I., Griffiths, G. & Blasco, R. (2000). Intracellular localization of vaccinia virus extracellular enveloped virus envelope proteins individually expressed using a Semliki Forest virus replicon. J Virol 74, 10535–10550. 11044098 10.1128/jvi.74.22.10535-10550.2000 PMC110928

[r17] Mathew, E., Sanderson, C. M., Hollinshead, M. & Smith, G. L. (1998). The extracellular domain of vaccinia virus protein B5R affects plaque phenotype, extracellular enveloped virus release, and intracellular actin tail formation. J Virol 72, 2429–2438. 9499104 10.1128/jvi.72.3.2429-2438.1998 PMC109543

[r18] Mathew, E. C., Sanderson, C. M., Hollinshead, R. & Smith, G. L. (2001). A mutational analysis of the vaccinia virus B5R protein. J Gen Virol 82, 1199–1213. 11297695 10.1099/0022-1317-82-5-1199

[r19] McIntosh, A. A. & Smith, G. L. (1996). Vaccinia virus glycoprotein A34R is required for infectivity of extracellular enveloped virus. J Virol 70, 272–281. 8523536 10.1128/jvi.70.1.272-281.1996 PMC189814

[r20] Olivari, S. & Molinari, M. (2007). Glycoprotein folding and the role of EDEM1, EDEM2 and EDEM3 in degradation of folding-defective glycoproteins. FEBS Lett 581, 3658–3664. 17499246 10.1016/j.febslet.2007.04.070

[r21] Parkinson, J. E. & Smith, G. L. (1994). Vaccinia virus gene A36R encodes a *M*_r_ 43–50 K protein on the surface of extracellular enveloped virus. Virology 204, 376–390. 8091668 10.1006/viro.1994.1542

[r22] Perdiguero, B., Lorenzo, M. M. & Blasco, R. (2008). Vaccinia virus A34 glycoprotein determines the protein composition of the extracellular virus envelope. J Virol 82, 2150–2160. 18094186 10.1128/JVI.01969-07 PMC2258926

[r23] Pütz, M. M., Alberini, I., Midgley, C. M., Manini, I., Montomoli, E. & Smith, G. L. (2005). Prevalence of antibodies to vaccinia virus after smallpox vaccination in Italy. J Gen Virol 86, 2955–2960. 16227216 10.1099/vir.0.81265-0

[r24] Roberts, K. L. & Smith, G. L. (2008). Vaccinia virus morphogenesis and dissemination. Trends Microbiol 16, 472–479. 18789694 10.1016/j.tim.2008.07.009

[r25] Roberts, K. L., Breiman, A., Carter, G. C., Ewles, H. A., Hollinshead, M., Law, M. & Smith, G. L. (2009). Acidic residues in the membrane-proximal stalk region of vaccinia virus protein B5 are required for glycosaminoglycan-mediated disruption of the extracellular enveloped virus outer membrane. J Gen Virol 90, 1582–1591. 19264647 10.1099/vir.0.009092-0 PMC2885056

[r26] Roper, R. L., Payne, L. G. & Moss, B. (1996). Extracellular vaccinia virus envelope glycoprotein encoded by the A33R gene. J Virol 70, 3753–3762. 8648710 10.1128/jvi.70.6.3753-3762.1996 PMC190251

[r27] Rottger, S., Frischknecht, F., Reckmann, I., Smith, G. L. & Way, M. (1999). Interactions between vaccinia virus IEV membrane proteins and their roles in IEV assembly and actin tail formation. J Virol 73, 2863–2875. 10074134 10.1128/jvi.73.4.2863-2875.1999 PMC104044

[r28] Sanderson, C. M., Frischknecht, F., Way, M., Hollinshead, M. & Smith, G. L. (1998). Roles of vaccinia virus EEV-specific proteins in intracellular actin tail formation and low pH-induced cell-cell fusion. J Gen Virol 79, 1415–1425. 9634084 10.1099/0022-1317-79-6-1415

[r29] Shida, H. (1986). Nucleotide sequence of the vaccinia virus hemagglutinin gene. Virology 150, 451–462. 3008418 10.1016/0042-6822(86)90309-0

[r30] Smith, G. L., Vanderplasschen, A. & Law, M. (2002). The formation and function of extracellular enveloped vaccinia virus. J Gen Virol 83, 2915–2931. 12466468 10.1099/0022-1317-83-12-2915

[r31] Takahashi-Nishimaki, F., Funahashi, S., Miki, K., Hashizume, S. & Sugimoto, M. (1991). Regulation of plaque size and host range by a vaccinia virus gene related to complement system proteins. Virology 181, 158–164. 1994573 10.1016/0042-6822(91)90480-y

[r32] Turner, P. C. & Moyer, R. W. (2006). The cowpox virus fusion regulator proteins SPI-3 and hemagglutinin interact in infected and uninfected cells. Virology 347, 88–99. 16378629 10.1016/j.virol.2005.11.012

[r33] Wagenaar, T. R. & Moss, B. (2007). Association of vaccinia virus fusion regulatory proteins with the multicomponent entry/fusion complex. J Virol 81, 6286–6293. 17409143 10.1128/JVI.00274-07 PMC1900102

[r34] Ward, B. M. & Moss, B. (2000). Golgi network targeting and plasma membrane internalization signals in vaccinia virus B5R envelope protein. J Virol 74, 3771–3780. 10729152 10.1128/jvi.74.8.3771-3780.2000 PMC111886

[r35] Wolffe, E. J., Isaacs, S. N. & Moss, B. (1993). Deletion of the vaccinia virus B5R gene encoding a 42-kilodalton membrane glycoprotein inhibits extracellular virus envelope formation and dissemination. J Virol 67, 4732–4741. 8331727 10.1128/jvi.67.8.4732-4741.1993 PMC237859

[r36] Wolffe, E. J., Katz, E., Weisberg, A. & Moss, B. (1997). The A34R glycoprotein gene is required for induction of specialized actin-containing microvilli and efficient cell-to-cell transmission of vaccinia virus. J Virol 71, 3904–3915. 9094667 10.1128/jvi.71.5.3904-3915.1997 PMC191542

